# Mechanisms Predisposing Penile Fracture and Long-Term Outcomes on Erectile and Voiding Functions

**DOI:** 10.1155/2014/768158

**Published:** 2014-04-13

**Authors:** Leonardo O. Reis, Marcelo Cartapatti, Rafael Marmiroli, Eduardo Jeronimo de Oliveira Júnior, Ricardo Destro Saade, Adriano Fregonesi

**Affiliations:** School of Medical Sciences, University of Campinas (UNICAMP) and Pontifical Catholic University of Campinas (PUC), PUC-Campinas, Rua Tessália Vieira de Camargo 126, Cidade Universitária “Zeferino Vaz,” 13083-887 Campinas, SP, Brazil

## Abstract

*Purpose.* To determine the mechanisms predisposing penile fracture as well as the rate of long-term penile deformity and erectile and voiding functions. *Methods.* All fractures were repaired on an emergency basis via subcoronal incision and absorbable suture with simultaneous repair of eventual urethral lesion. Patients' status before fracture and voiding and erectile functions at long term were assessed by periodic follow-up and phone call. Detailed history included cause, symptoms, and single-question self-report of erectile and voiding functions. *Results.* Among the 44 suspicious cases, 42 (95.4%) were confirmed, mean age was 34.5 years (range: 18–60), mean follow-up 59.3 months (range 9–155). Half presented the classical triad of audible crack, detumescence, and pain. Heterosexual intercourse was the most common cause (28 patients, 66.7%), followed by penile manipulation (6 patients, 14.3%), and homosexual intercourse (4 patients, 9.5%). “Woman on top” was the most common heterosexual position (*n* = 14, 50%), followed by “doggy style” (*n* = 8, 28.6%). Four patients (9.5%) maintained the cause unclear. Six (14.3%) patients had urethral injury and two (4.8%) had erectile dysfunction, treated by penile prosthesis and PDE-5i. No patient showed urethral fistula, voiding deterioration, penile nodule/curve or pain. *Conclusions.* “Woman on top” was the potentially riskiest sexual position (50%). Immediate surgical treatment warrants long-term very low morbidity.

## 1. Introduction

Penile fracture is a relatively uncommon clinical condition that frequently causes fear and embarrassment for the patient, hypothetically resulting in delayed search for medical assistance, which can lead to an impairment of sexual and voiding functions [1]. Its incidence and etiologies vary according to geographic region, sexual behavior, marital status, and culture.

Considering that most studies are retrospective and based on patients' records, information regarding the social dynamics surrounding penile fracture is scarce in the literature, mainly concerning the most potentially risky sexual position. Kramer pioneering work demonstrated an association between penile fracture and sexual intercourse under stressful situation, but no further information was provided [2].

This study aims to dissect potential risk factors related to penile fracture occurrence. Additionally, preoperative evaluation, surgical management, and its association with long-term sexual and voiding functions were evaluated.

## 2. Patient and Methods

Between January 2000 and March 2013, all patients that presented at three emergency hospitals responsible for most of the surgical emergencies in a metropolitan region consisting of over 3 million inhabitants (University of Campinas (UNICAMP), Pontifical Catholic University of Campinas (PUC), Campinas, and Mario Gatti Municipal Hospital (HMMG)) with clinical suspicion of penile fracture were included in this study, following the best ethics criteria according to the local ethical committees.

A surgeon at the emergency room first evaluated all patients, and a second evaluation by a urologist was a mandatory request.

All data were collected from patients' records of the three departments and by phone interview, considering the following aspects: detailed history including symptoms, type of relationship (homosexual/heterosexual), mechanism of trauma, sexual position (when applied), clinical findings at physical examination, imaging results (when requested by the clinical judgment of the urologist), presence of urethral injury, outcomes, and long-term complications regarding sexual and voiding functions. A single-question self-report of erectile and voiding functions was used for all cases with 2 possible answers: normal and abnormal.

If urethral injury was suspected (urethral bleeding or urinary retention), evaluation by retrograde urethrogram was performed, without delaying surgical approach. Doppler Ultrasound was performed in patients with unclear history of trauma or poor clinical findings on physical examination.

All patients were managed on an emergency basis by the urologist on duty via immediate subcoronal circumferential degloving incision. The defect of the tunica albuginea was closed by 3.0 polyglactin 910 sutures. In case of concomitant urethral lesion, the defect was repaired simultaneously by primary absorbable 4.0 polydioxanone (PDS) sutures and urethral catheterization for 10 days.

Intraoperative findings were matched with imaging test, and the following aspects were considered: localization and size of rupture and presence or absence of urethral injury. Voiding and erectile functions were evaluated at long term by periodic follow-up and phone call. Patients' status before penile fracture was assessed retroactively.

Forty-four patients were admitted at the three emergency centers with the suspicion of penile fracture, of which 42 (95.45%) had the condition confirmed after clinical, radiological, and surgical evaluation. The mean age was 34.5 years (range: 18–60). Half of patients (*n* = 21, 50%) presented with the classical triad of an audible crack followed by detumescence and pain. The presentation time of patients to the hospital after penile fracture ranged from 0.5 to 6 hours. The mean follow-up after penile trauma was 59.3 months (range: 9–155).

## 3. Results

Table 1 shows detailed clinical findings. Heterosexual intercourse was the most common cause of fracture (28 patients, 66.7%), followed by penile manipulation (6 patients, 14.3%) and homosexual intercourse (4 patients, 9.5%). Four patients (9.5%) opted to maintain the cause unclear.

Regarding sexual position reported by man in heterosexual relationship (*n* = 28), “woman on top” was the most common situation associated, corresponding with 14 cases (50%), followed by “doggy style” in 8 cases (28.6%). Six patients (21.4%) reported being on top at the moment of injury. Twenty-six (92.9%) patients reported vaginal penetration, and only 2 (7%) confirmed anal penetration.

Regarding homosexual intercourses, half of the patients (*n* = 2) reported being on top and the other half (*n* = 2) had his partner on “doggy style” position.

Overall, sixteen patients (36.4%) had preoperative ultrasound, with a positive predictive value of 87.5%. The two cases of mismatch between the ultrasound image and intraoperative findings had a lesion smaller than 2 mm reported by the radiologist, which were not confirmed by surgery.

Four (9.5%) tunica albuginea tears were bilateral with an overall mean size of 1.6 cm and six (14.3%) patients had urethral injury diagnosed intraoperatively, with 5 being previously confirmed with retrograde urethrogram due to urethral bleeding. There was no correlation between injury extension and mechanism (sexual position, etc.).

At last follow-up, no patient showed urethral fistula, deterioration of voiding, penile nodule, penile pain during intercourse, or clinically significant penile deviation. Only two (4.8%) patients had erectile dysfunction, one of them treated by penile prosthesis and another with PDE-5 inhibitor, and four (9.5%) patients presented minimal penile curvature without clinical impact, characterized as minor negligible curvature, not impairing penetration, usually <20°.

## 4. Discussion

The rupture of the tunica albuginea of the corpora cavernosa defines penile fracture that occurs with the organ in an erectile position. Diagnosis is made by history and clinical examination, and the classic triad of an audible “cracking” sound, followed by immediate detumescence and pain, is usually present. Although imaging may be required for better evaluation, usually it is unnecessary [3]. Urethral bleeding and voiding incapacity can be an alert to urethral injury and a retrograde urethrogram should promptly be requested to optimize treatment planning with simultaneous urethral repair during surgery [4].

Due to its protected location and relative mobility, injuries to the flaccid penis are scarce and with diverse mechanisms such as penetrating and degloving or amputation injuries to the flaccid penis and are beyond the scopes of this study.

The present study established for the first time in the literature the relation between sexual position and penile fracture, shedding light on its potential impact on the risk increment. Kramer in 2011 published a case series of 16 patients with penile fracture requiring surgery and an association between this clinical condition and sexual intercourse during stressful situations was verified [2]. In our report, we found it difficult to apply this information, mostly because a considerable number of patients preferred to keep obscure the circumstances involving the incident. Despite that, it was possible to verify that “woman on top” was the most frequent sexual position associated with penile fracture.

Our hypothesis is that when woman is on top she usually controls the movement with her entire body weight landing on the erect penis, not being able to interrupt it when the penis suffers a wrong way penetration, because the harm is usually minor in woman with no pain but major in the penis. On the contrary, when the man is controlling the movement, he has better chances of stopping the penetration energy in response to the pain related to the penis harm, minimizing it.

It is also important to emphasize that about one-fourth of patients (*n* = 10, 23.8%) gave no details about the circumstances involved in penile fracture: manipulation with no additional information was reported by 6 patients (14.3%) and 4 patients (9.5%) opted to maintain the cause unclear. This posture hinders the accurate understanding of factors surrounding penile fracture, mainly potential predisposing mechanisms.

Interestingly, while in Western society vaginal intercourse is the main cause, more than half of the reported penile fractures in the Middle East, especially in Iran, are inflated by manual bending of the erected penis to achieve detumescence due to cultural circumstances (i.e., forceful hiding of an erect penis in underwear, known as Taghaandan practice, “breaking the Qholenj”) [5].

Additionally, we showed excellent long-term outcomes in terms of maintenance of erectile and voiding functions, in a mean follow-up of 59.3 months (range: 9–155). Two patients reported erectile dysfunction, representing only 4.8% of cases, but only one needed surgical treatment, which is found similarly in the literature. Thus, our study supports that immediate surgical approach is the best treatment for clinically confirmed fractures compared to conservative management that can lead to erectile dysfunction in up to 50% of patients [6].

The use of ultrasound (US) as a diagnosis method is controversial, since anamnesis followed by rigorous physical examination is sufficient in most of the cases that present with a typical history [7, 8]. However, in some cases, these findings are so subtle that diagnosis can become unclear. In one study, Beysel et al. noticed US inaccuracy of 15% of patients in a series of thirteen cases [9]. In the present study, we demonstrated the same accuracy, with only 2 false positive US results in 16 patients.

We believe that immediate surgical correction is the best treatment and conservative posture is not an option in our centers. Actually, early surgery is the current standard of care because it accurately distinguishes false from true penile fracture, speeds recovery, and results in a smaller scar of the tunica albuginea, lessening the chances of subsequent erectile dysfunction and deformity to far less than 5–10%, compared to over 50% of morbidity after conservative management [6, 10].

If urethral injury is suspected, most authors advocate a preoperative retrograde urethrogram. Others advocate flexible cystoscopy in the operating room before inserting the Foley catheter [1]. It is our perception that surgical exploration is the gold standard to confirm and treat urethral injury and costly imaging methods should not delay surgical treatment in the acute setting. As corpus spongiosum injury almost always occurs at the same level of the corpora cavernosal injury, false negative results will ordinarily be recognized during early surgical exploration, avoiding the later urethral stricture.

The current work is not free of limitations. Although data were prospectively assessed by periodic follow-up visits in addition to phone calls to complete eventually missed facts, the study holds the limitations of a retrospective analysis, sharing the drawbacks of most studies on the issue. Also, data regarding erectile and voiding functions were obtained by single-question self-report, which was limited to only 2 possible answers: normal and abnormal, not quantifying a possible dysfunction.

Moreover, information concerning factors related to penile fracture is always obtained by the story that patients tell their doctors. Given the intimacy and taboos of patients' sexual life, while one-fourth preferred to omit details, many patients might have been imprecise about the real truth.

Striving to improve data quality, future protocols should systematically inform patients about the importance of accurate information on the subject and also about the precautions to keep their intimacy uncovered aiming at more reliable data.

## 5. Conclusions

Our study supports the fact that sexual intercourse with “woman on top” is the potentially riskiest sexual position related to penile fracture. Social dynamics surrounding penile fracture is still obscure and patients should systematically be informed about the importance of accurate information. Immediate surgical exploration warrants very low morbidity at even long follow-up.

## Figures and Tables

**Table 1:** Patients characteristics.

Variables	Years (range)
Age	34.5 (18–60)

Mechanism of trauma	*N* (%)

Sexual intercourse*	32 (76.2)
Woman on top^#^	14 (50)
“Doggy style”^#^	8 (28.6)
Man on top^#^	6 (21.4)
Penile manipulation	6 (14.3)
Unclear	4 (9.5)

Clinical findings	*N* (%)

Pain	38 (90.5)
Swelling	19 (45.2)
Cracking sound	21 (50)
Detumescence	34 (80.9)
Hematoma	39 (92.8)
Urethral bleeding	5 (11.9)

Long-term outcomes	Follow-up: 59.3 m (9–155)

Erectile dysfunction	2 (4.8)
Significant penile deviation	0
Voiding dysfunction	0

*Counting hetero- and homosexual intercourse cases (28 and 4, resp.).

^#^Among heterosexual intercourse cases (*n* = 28).
